# Molecular Basis for the Divergent Inhibition of α-Amylase and α-Glucosidase by Phenolic Acids: The Critical Role of Hydroxyl Substitution

**DOI:** 10.3390/foods15111972

**Published:** 2026-06-02

**Authors:** Shuang Yang, Yongxing Li, Weiyu Han, Wenhao Cao, Zhihui Hu, Zhangliang Zhu, Mei Li, Jianhui Feng, Jinfang Zhang

**Affiliations:** 1 School of Food Engineering, Ludong University, Yantai 264025, China; syang979797@163.com; 2 Center for Mitochondria and Healthy Aging, College of Life Sciences, Yantai University, Yantai 264005, China; liyongxing0826@163.com (Y.L.); hwy02282024@163.com (W.H.); wenhaocao2024@163.com (W.C.); huzhihui9968@163.com (Z.H.); jhfeng0122@163.com (J.F.); 3 Graduate School of Biostudies, Kyoto University, Kyoto 606-8501, Japan

**Keywords:** caffeic acid, p-hydroxycinnamic acid, α-amylase, α-glucosidase, inhibition patterns

## Abstract

The global rise in metabolic disorders demands novel interventions targeting starch digestion. This study investigated two dietary phenolic acids (caffeic acid (CA) and p-hydroxycinnamic acid (p-HA)) as inhibitors of α-amylase and α-glucosidase through integrated experimental and computational approaches. Molecular docking showed distinct binding modes, and CA formed stable hydrogen bonds with catalytic residues of α-glucosidase, while p-HA interacted mainly with α-amylase via hydrophobic contacts. Enzyme kinetics revealed concentration-dependent mixed-type inhibition, with CA being more potent against α-glucosidase and p-HA against α-amylase. Spectroscopic analysis indicated both acids induced structural changes in the enzymes, with CA causing greater α-helix reduction (Δ7.03% vs. Δ2.10%) by altering the tryptophan microenvironment. Moreover, both compounds significantly suppress glucose absorption in the proximal small intestine in an ex vivo everted gut sac model, with p-HA exhibiting exceptional efficacy in the duodenum. These findings clarify structure–activity relationships and support the potential use of CA and p-HA as local intestinal agents for modulating carbohydrate absorption.

## 1. Introduction

The escalating pandemic of metabolic syndrome, characterized by obesity-driven insulin resistance and dyslipidemia, currently affects approximately 1.9 billion adults globally [1,2]. Starch is the main source of carbohydrates consumed by the human body and is also one of the main raw materials for food production. Therefore, certain processing of starch to reduce its glycemic index has become a research hotspot. The two main digestive enzymes that play a major role in the digestion and absorption of starch are α-amylase and α-glucosidase. Therefore, inhibiting the activity of hydrolase is the main method to prevent rapid, drastic postprandial blood glucose fluctuations [3]. Clinically, acarbose, voglibose and miglitol are commonly used to delay and treat Type 2 diabetes (T2D). They mainly work by inhibiting the activity of carbohydrate hydrolase enzymes, reducing the hydrolysis rate of oligosaccharides and monosaccharides, thereby delaying the body’s absorption of sugar. However, these drugs generally have side effects such as abdominal distension, diarrhea, abdominal pain and gastrointestinal dyspepsia [4,5]. Therefore, searching for hydrolase inhibitors with less toxic side effects and better hypoglycemic effects from natural products is of great significance for the management of T2D.

Phytochemical-mediated enzyme inhibition has emerged as a strategic approach in metabolic disease management, with polyphenols demonstrating dual-phase bioactivity: primary prevention through ROS scavenging and secondary intervention via enzymatic modulation [6,7]. Phenolic acids, as a structurally distinct subclass of polyphenols characterized by aromatic rings with hydroxyl groups, exhibit particularly promising therapeutic potential [8]. Among these, hydroxycinnamic acids exhibit unique stereoelectronic properties-their conjugated π-system and ortho-dihydroxy configuration enable mixed-type inhibition enzyme binding through π-π stacking with catalytic residues, and stabilization of transition states via hydrogen-bond networks [9]. CA and p-HA are two of the most representative and widely distributed dietary hydroxycinnamic acids in the human daily diet, sharing an identical C6-C3 cinnamic acid backbone with the only structural difference in the number and position of hydroxyl substitutions on the benzene ring, which makes them ideal molecular probes to explore the structure–activity relationship of phenolic acids. Nevertheless, critical knowledge gaps persist regarding structure–inhibition relationships: (1) the molecular basis for the divergent inhibition of α-amylase and α-glucosidase by structurally similar hydroxycinnamic acids—specifically, how hydroxyl substitution patterns dictate binding selectivity between the two enzymes; (2) the direct link between enzyme conformational changes and intestinal glucose absorption inhibition, which has rarely been explored in parallel with enzyme inhibition; (3) the allosteric effects of hydroxylation patterns on enzyme conformational dynamics. Screening functional structures critical for enzyme inhibition and clarifying their inhibitory mechanisms can improve the development efficiency of starch hydrolase inhibitors and guide the processing and utilization of polyphenol-rich plants.

This study systematically investigates the inhibitory effects of these phenolic acids (CA and p-HA) on key carbohydrate-digesting enzymes (α-amylase and α-glucosidase) through an integrated experimental approach combining fluorescence quenching assays with Stern-Volmer analysis, molecular docking simulations, and ex vivo intestinal absorption modeling via the everted gut sac technique. These findings can provide new insights into the structure–activity relationship of polyphenols and guide the development of next-generation enzyme inhibitors with enhanced specificity and reduced side effects.

## 2. Materials and Methods

### 2.1. Experimental Materials

CA (CAS: 331-39-5) was purchased from Aladdin Reagent Co., Ltd. (Shanghai, China). p-HA (CAS: 7400-08-0) and p-nitrophenyl-α-D-glucopyranoside (pNPG) were bought from Shanghai Yuanye Biotechnology Co., Ltd. (Shanghai, China). α-amylase (porcine pancreas) (A3176, 10.3 U/mg) and α-glucosidase (G5003, 100 U) were purchased from Sigma-Aldrich Co., Ltd. (St Louis, MO, USA). 3, 5-dinitrosalicylic acid was sourced from Shanghai Macklin Biochemical Co., Ltd. (Shanghai, China). All other chemicals and reagents were of analytical grade.

### 2.2. Molecular Docking

Molecular docking simulations were conducted using AutoDock Vina 1.5.3 (The Scripps Research Institute) to investigate ligand–enzyme interactions. Docking protocol was validated by redocking the co-crystallized ligand of α-amylase (PDB: 1PIF) and α-glucosidase (PDB: 5ZCB) into the respective active pockets; the root-mean-square deviation (RMSD) between the redocked pose and the original co-crystallized pose was <2.0 Å, confirming the reliability of the docking parameters. The crystal structures of α-amylase (PDB ID: 1PIF) and α-glucosidase (PDB ID: 5ZCB) were retrieved from the RCSB Protein Data Bank (http://www.rcsb.org, accessed on 30 March 2026). Protein preparation involved: (i) removal of water molecules and heteroatoms via PyMOL 2.5.0, (ii) addition of polar hydrogen atoms, and (iii) assignment of Gasteiger partial charges. Ligand structures of CA (CID: 689043) and p-HA (CID: 637542) were acquired from PubChem (https://pubchem.ncbi.nlm.nih.gov, accessed on 30 March 2026) and energy-minimized using the UFF force field in PyMOL [10]. The grid box was set to 50 × 50 × 50 (x = 34.0, y = 22.4, z = 48.5) to fully cover the catalytic pocket and potential allosteric sites of the target enzymes, ensuring comprehensive sampling of plausible binding modes without excessive computational cost, setting exhaustiveness to 50 while maintaining default parameters for other variables. A total of 20 independent docking poses were generated for each ligand–enzyme complex. The optimal binding pose was selected based on the following criteria: (1) the lowest binding free energy (ΔG); (2) formation of stable hydrogen bonds and hydrophobic interactions with key catalytic residues; (3) binding orientation matching the enzyme active pocket and blocking the substrate binding channel. Resultant binding poses were visualized in PyMOL, with key interaction parameters including binding free energy (ΔG, kcal/mol) and hydrogen bond distances extracted from the top-ranked conformation. Docking provides qualitative information about binding poses and interaction types, not quantitative evidence of inhibitory activity.

### 2.3. Determination of Starch-Digesting Enzyme Activity

#### 2.3.1. Inhibitory Activity of Polyphenols Against α-Amylase

The activity of α-amylase (7 U/mL) was determined according to the method of Chen et al. [11]. The enzymatic reaction system contained 100 μL of α-amylase combined with 100 μL of test compounds (CA or p-HA at specified concentrations). Following 5 min pre-incubation at 37 ± 0.5 °C in a thermostatic water bath, the enzymatic reaction was initiated by adding 800 μL of pre-warmed (37 °C) soluble starch solution (1% *w*/*v* in buffer). The reaction mixture was maintained at 37 °C for precisely 10 min before termination through the addition of 1 mL DNS (3,5-Dinitrosalicylic acid, 10 g/L) reagent. After boiling the terminated reactions for 10 min in a water bath and cooling to ambient temperature (25 ± 2 °C), absorbance (A) measurements were recorded at 540 nm. The half-inhibition concentration (IC_50_ value) was obtained by regression analysis of the enzyme activity inhibition rate-sample concentration curve. Enzyme inhibition activity was expressed as the inhibition ratio and calculated using the following formula [11]:

(1) Inhibition (%) = [1 − (*A_sample_* − *A_control_*)/(*A_blank_* − *A_(blank-control)_*] × 100%

where *A_sample_* is the absorbance value of the sample group; *A_control_* is the absorbance value of the control group; *A_blank_* is the absorbance value of the blank group; *A*_(*blank−control*)_ is the difference in absorbance values between the blank group and the control group.

#### 2.3.2. Inhibitory Activity of Polyphenols Against α-Glucosidase

The α-glucosidase (5 U/mL) inhibitory activity was quantified using a modified pNPG hydrolysis assay based on Fan et al. [12]. In brief, reaction mixtures containing 20 μL α-glucosidase solution, 20 μL of serially diluted phenolic acid inhibitors (CA or p-HA), and 90 μL phosphate-buffered saline (PBS, 0.1 M, pH 6.8) were pre-incubated in 96-well microplates at 37 °C for 5 min. The enzymatic reaction was initiated by adding 20 μL pNPG substrate (1 mM), followed by incubation at 37 °C for precisely 20 min. The reaction was terminated by adding 50 μL sodium carbonate solution (0.2 M). After cooling to room temperature (25 ± 1 °C), the absorbance at 405 nm was measured. The enzyme inhibition activity was calculated using Equation (1).

### 2.4. Inhibition Kinetics of Polyphenols on Digestive Enzymes

Enzyme solutions (α-amylase and α-glucosidase), varying substrate concentrations (starch: 10 mg/mL; pNPG: 1 mM), and polyphenol solutions at different concentrations were prepared. Enzyme activity assays were performed according to the methods described in Section 2.3.1. (for α-amylase) and Section 2.3.2. (for α-glucosidase). To determine the type of enzyme inhibition exerted by the phenolic acids, Lineweaver-Burk plots (1/v vs. 1/[S]) were constructed and analyzed.

### 2.5. Fluorescence Quenching Analysis of Phenolic Acid-Digestive Enzyme Interactions

Fluorescence quenching experiments were conducted to investigate the molecular interactions between phenolic acids and digestive enzymes, adopting a modified methodology based on Wang et al. [13]. Enzyme solutions (0.7 U/mL or 0.5 U/mL) were pre-incubated with serially diluted polyphenol solutions for 10 min to establish binding equilibrium before spectral measurement. Fluorescence emission spectra were acquired using a Cary Eclipse fluorometer (Shimadzu, Kyoto, Japan) across three temperatures (298, 304, and 310 K), with excitation at 280 nm and emission scanning from 300 to 400 nm. All measurements were corrected for background fluorescence using buffer blanks. Key parameters including the Stern-Volmer constant (K*_sv_*), quenching rate constant (K*_q_*), binding constant (K_a_), and quenching mechanism, were determined through Stern-Volmer equation analysis.

The fluorescence quenching mechanism can be classified into two primary types: dynamic quenching and static quenching. Dynamic quenching occurs through collisional encounters between the quencher and fluorophore, with the quenching efficiency following the linear relationship described by the Stern-Volmer equation. Static quenching, in contrast, results from ground-state complex formation between the molecules.

(2) F_0_/F = 1 + *K_q_τ*_0_[*Q*] = 1 + *K_sv_* [*Q*]

Static quenching is a process in which the interaction between the quencher and the fluorescent substance forms a ground-state quencher-fluorophore complex. The same quencher can produce dynamic quenching and static quenching at the same time. At this time, the Stem-Volmer diagram bends upward, and the concave surface faces the y-axis. The quenching process follows the modified Stem-Volmer equation.

(3) log(F_0_/F − 1) = *nlog*[*Q*] + *logK_a_*

In addition to the above situation, another quenching that may occur is the distance effect, that is, the quencher and the fluorescent group do not produce the ground state complex, nor do they collide, but the fluorescence quenching occurs within a certain range around the quencher. It is expressed by Stem-Volmer’s modified equation.

(4) logK*_a_* = − (Δ*H*/2.303*R*) × (1/*T*) + (Δ*S*/2.303*R*)

(5) ΔG = Δ*H* − *T*Δ*S*

### 2.6. UV-Visible Absorption Spectroscopy Analysis

For UV-vis spectral analysis, enzyme solution (α-amylase (0.7 U/mL) or α-glucosidase (0.5 U/mL)) was mixed with an equal volume of either CA or p-HA solution. The mixture was incubated at 37 °C for 10 min to allow sufficient interaction between the enzymes and phenolic acids. Subsequently, UV-vis absorption spectra were recorded from 200 to 400 nm using a spectrophotometer (UV-5100). All spectral data were corrected by subtracting the background absorption of the solvent control.

### 2.7. Circular Dichroism (CD) Spectroscopy

CD spectroscopy was carried out on a JASCO J-1500 CD spectrometer (Tokyo, Japan). Briefly, α-amylase (0.7 U/mL) or α-glucosidase (0.5 U/mL) was mixed with the phenolic acid and incubated at 298 K for 10 min. Spectra were then acquired over the wavelength range of 200–250 nm, with the background signal from PBS buffer subtracted [14]. The secondary structure composition (α-helix, β-sheet, β-turn, and random coil) of the proteins was calculated using the CDNN 2.1 software.

### 2.8. Everted Intestinal Sac Preparation and Sampling

The everted gut sac model was established following an adapted methodology based on reference [15]. Sprague-Dawley (SD) rats (200 ± 20 g, male) were obtained from Jinan Pengyue Experimental Animal Breeding Co., Ltd. (Jinan, China), and acclimatized for one week (23 ± 2 °C, 12 h light-dark cycle). A total of 9 male SD rats were used in this experiment, which were randomly divided into three groups (*n* = 3 per group): control group, CA treatment group, and p-HA treatment group. All samples were collected from different tissues of the same individual animal. All animal procedures were approved by the Ethical Committee for the Experimental Use of Animals, Yantai University (YDLL2024R053).

Before the experiment, the rats were fasted for 12 h and had free access to water. Upon achieving surgical anesthesia, duodenal, jejunal, ileal and colonic segments (4 cm each) were immediately excised and immersed in oxygenated Tyrode’s solution (37 °C) to remove luminal contents. Mesenteric attachments and adipose tissue were carefully dissected away. Each intestinal segment was everted using a glass rod to position the serosal surface inward, and cannulated at both ends. The sac was filled with 3 mL pre-warmed Tyrode’s solution (pH 7.4) and suspended in oxygenated Tyrode’s solution containing test compounds (CA/p-HA at specified concentrations,1 mg/mL), maintained at 37 °C under continuous carbogen (95% O_2_/5% CO_2_) gassing [16]. Samples were collected at different time points (15, 30, 45, 60, 90, 120 and 180 min), with immediate replenishment of equal volumes of fresh Tyrode’s solution to maintain constant volume. The glucose content was determined using the DNS method. Samples were stored at −80 °C until analysis.

### 2.9. Statistical Analysis

Data analysis was carried out using Origin 2021 software (Origin Lab, Northampton, MA, USA) and IBM SPSS Statistics (v27.0, IBM, Chicago, IL, USA). Each experiment was performed in triplicate and results were described as mean values. The significant difference was analyzed using analysis of variance (ANOVA) with a significance level of 5% (*p* < 0.05).

## 3. Results

### 3.1. Molecular Docking

**Figure 1:**
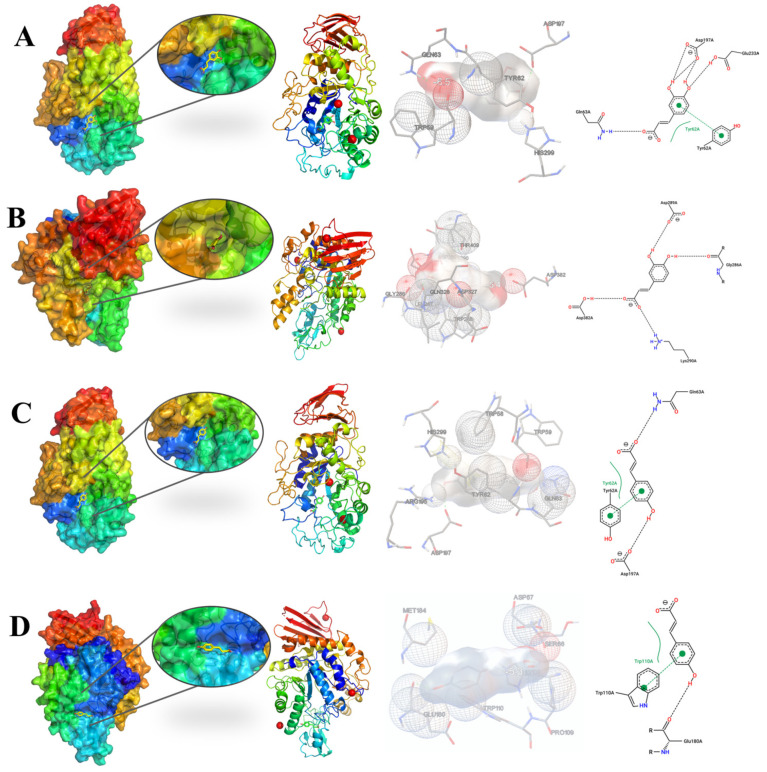
Molecular docking reveals the effects of caffeic acid (**A**,**B**) and p-hydroxycinnamic acid (**C**,**D**) on α-amylase (**A**,**C**) and α-glucosidase (**B**,**D**).

Molecular docking simulations were performed to characterize the qualitative binding modes of CA and p-HA with α-amylase and α-glucosidase. Residues Asp-197, Glu-233, and Asp-300 in α-amylase, and analogous regions in α-glucosidase, are critical for catalytic activity; thus, exploring inhibitor binding here helps determine their potential as active-site-directed agents. The docking results revealed that both CA and p-HA bind within hydrophobic pockets in the active site regions of the enzymes (Figure 1, Table S1). CA exhibited binding energies of −6.5 kcal/mol for α-amylase and −5.4 kcal/mol for α-glucosidase, interacting with key residues including Gln-63, GLU-233, Tyr-62, Asp-197, and His-299 in α-amylase, and Asp-382, Asp-289, Gly-286, and Lys-290 in α-glucosidase. Similarly, p-HA showed binding energies of −6.2 kcal/mol with α-amylase and −5.3 kcal/mol with α-glucosidase, forming interactions with residues such as Gln-63, Asp-197, and Tyr-62 in α-amylase, and Glu-180 and Trp-110 in α-glucosidase. These values fall within the weak-to-moderate binding energy range typical for dietary polyphenols and only reflect qualitative binding preferences rather than quantitative inhibitory strength. The structural differences between CA and p-HA contribute to their differential binding affinity and inhibitory trend. CA possesses ortho-diphenolic hydroxyl groups (at C3 and C4), which facilitate dual hydrogen bonding and π-π stacking interactions with complementary residues in the enzyme active sites [17]. This structural feature tends to enhance binding stability, particularly with α-glucosidase, which aligns with its stronger inhibitory activity observed in IC_50_ and kinetic assays. In contrast, p-HA contains a single para-hydroxyl group, which reduces overall molecular polarity and increases hydrophobic compatibility. This may favor its interaction with the predominantly hydrophobic subpockets within α-amylase, consistent with its relatively higher inhibitory potency toward α-amylase [18,19].

This is the first study to systematically clarify that hydroxyl substitution is the core determinant of the divergent inhibition selectivity of hydroxycinnamic acids toward α-amylase and α-glucosidase. The ortho-dihydroxyl configuration in CA promotes stronger, multi-dentate hydrogen bonding and π–π stacking with polar catalytic residues of α-glucosidase, leading to selective and potent α-glucosidase inhibition. Conversely, the single para-hydroxyl group of p-HA enhances hydrophobic compatibility, driving preferential binding to the hydrophobic subpocket of α-amylase, resulting in selective α-amylase inhibition. This differential binding mechanism directly explains the opposite inhibitory selectivity of the two structurally similar phenolic acids, which is a novel finding not reported in previous studies. These findings provide a rational basis for understanding the structure–activity relationship and highlight the potential for optimizing phenolic acid derivatives as selective enzyme inhibitors.

### 3.2. Effects of Phenolic Acids on Starch Digestive Enzyme Activities

**Figure 2:**
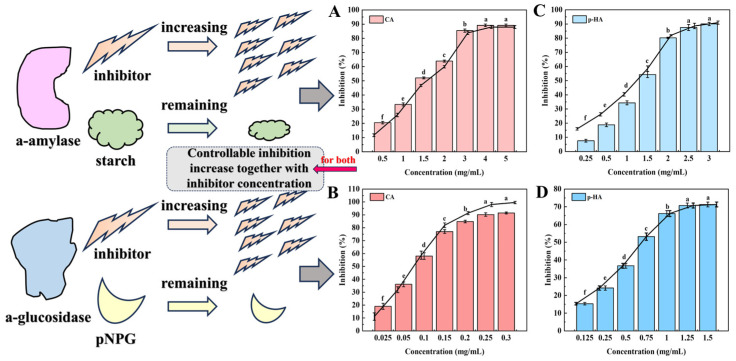
Inhibitory effects of caffeic acid (CA) and p-hydroxycinnamic acid (p-HA) on α-amylase (**A**,**C**) and α-glucosidase (**B**,**D**) activities. Different letters indicate statistical differences (*p* < 0.05).

The median inhibitory concentration (IC_50_) is a common indicator for evaluating the inhibitory activity of compounds against α-amylase. As shown in Figure 2, both CA and p-HA exhibited concentration-dependent inhibition of α-amylase and α-glucosidase, with their inhibitory effects increasing at higher mass concentrations. Both polyphenols exhibited moderate to good inhibitory activity against α-amylase (IC_50_ = 1.32 ± 0.15 mg/mL and 1.12 ± 0.25 mg/mL for CA and p-HA, respectively) and α-glucosidase (IC_50_ = 0.07 ± 0.01 mg/mL and 0.64 ± 0.18 mg/mL, respectively). However, for α-glucosidase, the inhibitory rate of acarbose at a concentration of 8.0 milligrams per milliliter reached 80%, which was higher than the IC_50_ values of CA and p-HA [9]. The inhibition of α-glucosidase was stronger than that of α-amylase for both compounds. p-HA demonstrated a stronger inhibitory effect on α-amylase than CA, whereas CA was more potent against α-glucosidase. It is important to note that IC_50_ values can be influenced by factors such as enzyme concentration and source, substrate type, reaction time, temperature, and pH [20]. According to literature reports, the inhibition of polyphenols is related to hydrogen bonding and conjugated π-system interactions [21,22,23]. Additionally, the selected phenolic acids feature a C-C bond conjugated to a carbonyl group, facilitating electron delocalization within the benzene ring. Variations in the number of hydroxyl (-OH) and carboxyl (-COOH) groups among these molecules also provide diverse structural motifs for potential enzyme interactions. Consequently, the structure–activity relationships underlying phenolic acid-enzyme interactions warrant further detailed investigation [21,22,23].

### 3.3. Kinetics Inhibition of Phenolic Acids Against α-Amylase and α-Glucosidase

**Figure 3:**
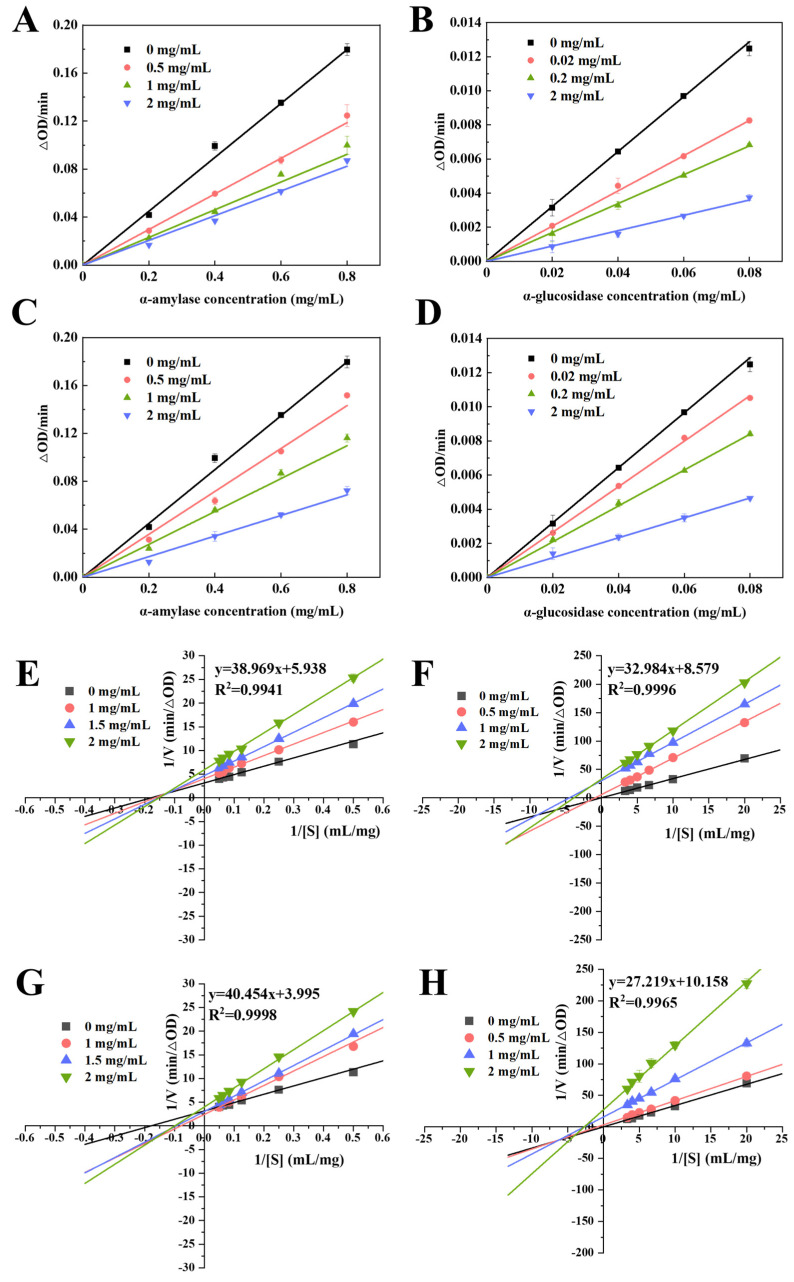
Reversible test (**A**–**D**) and Lineweaver–Burk plots (**E**–**H**) of polyphenols against α-amylase (**A**,**C**,**E**,**G**) and α-glucosidase (**B**,**D**,**F**,**H**). ((**A**,**B**,**E**,**F**): caffeic acid; (**C**,**D**,**G**,**H**): p-hydroxycinnamic acid).

Figure 3A–D shows the relationship curve between the reaction rate (v) and different concentrations of α-amylase/α-glucosidase. From the figure, it can be seen that after the addition of phenolic acids, all lines pass through the origin and exhibit decreasing slopes with increasing inhibitor concentration, confirming that inhibition by both phenolic acids is reversible and arises from non-covalent interactions [24].

Reversible inhibition includes competitive inhibition, non-competitive inhibition and mixed inhibition. To elucidate the inhibition mechanisms of CA and p-HA on α-amylase/α-glucosidase, Lineweaver-Burk plots were used to determine inhibition types and kinetic parameters (Figure 3E–H). For α-amylase inhibition (Figure 3E,G), the regression lines for both polyphenols intersected in the second quadrant. These observations collectively demonstrate mixed inhibition for both compounds against α-amylase, consistent with previous reports for CA [25]. For α-glucosidase (Figure 3F,H), the system with the addition of CA and p-HA intersects in the third quadrant. Therefore, it can be concluded that the inhibition types of both phenolic acids on α-glucosidase belong to the mixed-type inhibition.

The inhibition constant (K*_i_*) is an important parameter for evaluating the binding capacity between digestive enzymes and inhibitors. A lower K_i_ value indicates a stronger binding affinity between the inhibitor and the digestive enzyme [23]. The type of inhibition exerted by phenolic acids on digestive enzymes was determined by calculating the competitive (K*_ic_*) and non-competitive (K*_iu_*) inhibition constants. As shown in Table 1, the K*_ic_* values of the two phenolic acids were both lower than their K*_iu_* values, indicating that competitive inhibition predominates in the mixed-type inhibition. This suggests that phenolic acids have a stronger binding ability with free amylase than with the phenolic acid-enzyme-substrate complex. The good linear fit of the slope curve indicates that there is only one or one class of binding sites between phenolic acids and digestive enzymes. Similar results were reported by Zheng et al., who found that chlorogenic acid acts as a mixed-type inhibitor of α-amylase, with a single category or single inhibition site on the enzyme [26]. Meanwhile, the order of the K*_ic_* values of the phenolic acids was consistent with that of the IC_50_ values. Both phenolic acids exhibited better inhibitory effects on α-glucosidase, with CA showing stronger inhibition. This indicates that the tighter the binding between phenolic acids and the enzyme’s active site, the higher their inhibitory activity. This finding contrasts with literature reporting competitive inhibition for other phenolic acids against α-glucosidase, such as persimmon tannin and gallic acid [23]. It has been confirmed that CA and p-HA exhibit mixed inhibition towards both α-amylase and α-glucosidase, and the competitive inhibition constant K_*ic*_ is always smaller than the non-competitive inhibition constant K*_iu_*. This result indicates that the affinity of these two phenolic acids for the free enzyme active sites is significantly higher than that for the allosteric sites of the enzyme-substrate complex. The observed discrepancies likely arise from structural variations among phenolic acid inhibitors and methodological factors influencing inhibition type determination, including inhibitor chemical properties, substrate concentration, and assay conditions [26]. Consequently, inhibition mechanisms exhibit significant compound-specific variation, even for the same target enzyme.

### 3.4. Fluorescence Spectra of Phenolic Acids Against α-Amylase and α-Glucosidase

**Figure 4:**
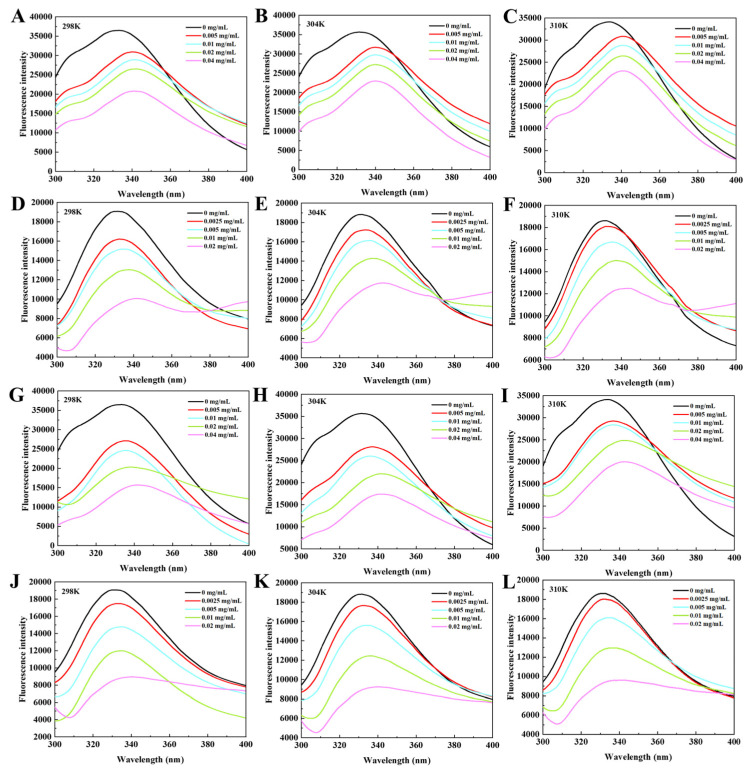
Effect of caffeic acid (**A**–**F**) and p-hydroxycinnamic acid (**G**–**L**) on the fluorescence spectra of α-amylase (**A**–**C**,**G**–**I**) and α-glucosidase (**D**–**F**,**J**–**L**).

The fluorescence spectra of CA in the presence of α-amylase and α-glucosidase are presented in Figure 4A–F. Upon excitation at 280 nm, the intrinsic fluorescence emission peaks of native α-amylase and α-glucosidase were observed near 348 nm and 336 nm, respectively. This intrinsic fluorescence primarily originates from tryptophan (Trp), tyrosine (Tyr), and phenylalanine (Phe) residues within the enzymes, owing to the chromophore groups on their side chains [27]. Upon interaction with CA, the intrinsic fluorescence intensity of both enzymes decreased significantly in a concentration-dependent manner, indicative of fluorescence quenching. Conversely, the quenching effect diminished with increasing temperature. This quenching phenomenon is likely induced by the formation of a complex between CA and the enzymes. Furthermore, CA induced a red shift in the fluorescence spectra of both enzymes. The maximum emission wavelengths of α-amylase (0.04 mg/mL) and α-glucosidase (0.02 mg/mL) were red-shifted by approximately 8 nm. This shift suggests that CA alters the hydrophobic microenvironment surrounding the amino acid residues within the starch-digestive enzymes.

These values are presented in the form of mean ± standard deviation (SD). The same letters indicate no significant difference within the same column (*p* < 0.05). IC_50_, half-maximal inhibitory concentration; V_max_, maximum hydrolysis rate; K_m_, Michaelis–Menten constant; in mixed inhibition, K_m_ can increase or decrease based on inhibitor affinity; K_ic_, competitive inhibition constant; K_iu_, uncompetitive inhibition constant; K_i_ is inversely proportional to binding affinity (lower K_i_ = stronger binding); CA, caffeic acid; p-HA, p-hydroxycinnamic acid.

**Figure 5:**
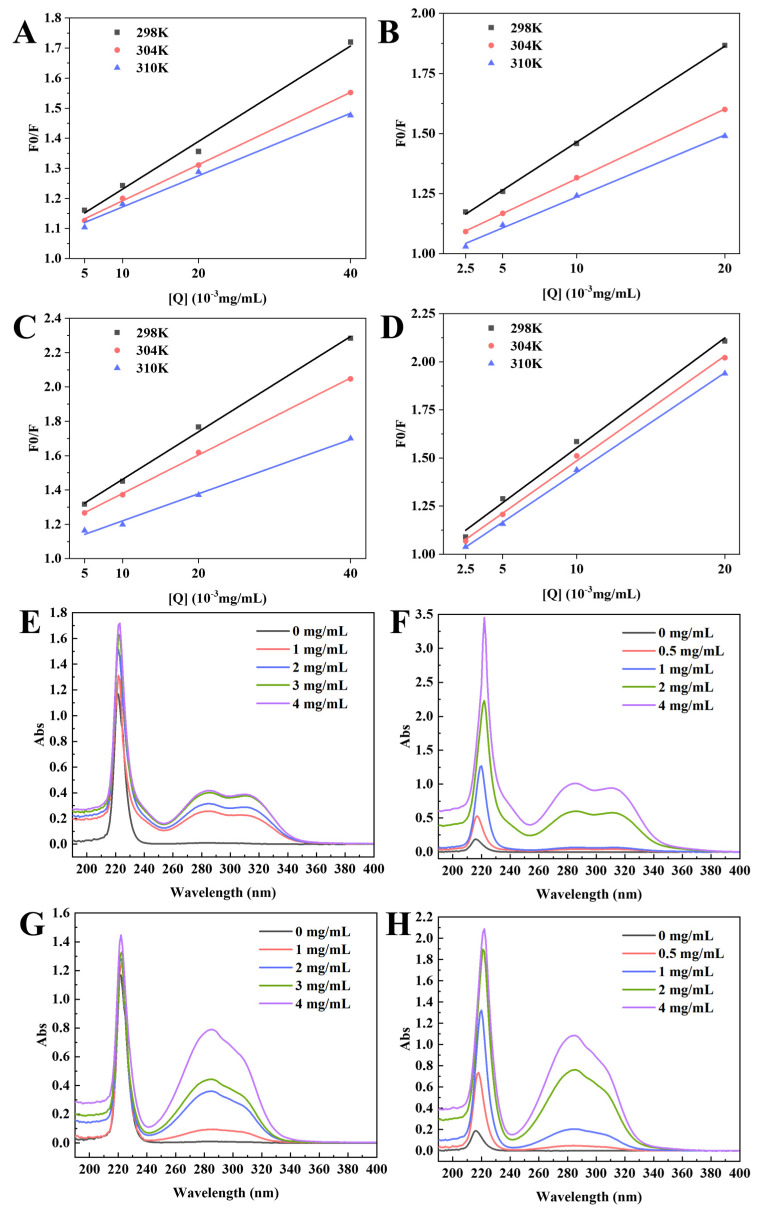
Effect of caffeic acid and p-hydroxycinnamic acid on the Stern-Volmer equation (**A**–**D**) and UV-vis absorption (**E**–**H**) spectra of α-amylase (**A**,**C**,**E**,**G**) and α-glucosidase (**B**,**D**,**F**,**H**). ((**A**,**B**,**E**,**F**): caffeic acid; (**C**,**D**,**G**,**H**): p-hydroxycinnamic acid).

As the concentration of phenolic acid compounds increases, the curve shows a good linear relationship (R^2^ > 0.99, Figure 5A–D), indicating that there is only a single type of fluorescence quenching mechanism between phenolic acid compounds and digestive enzymes [28]. The fluorescence quenching mechanism usually includes static quenching and dynamic quenching. The static quenching is caused by the combination of the ground state protein and the quencher to form a non-fluorescent complex, while the dynamic quenching is caused by the collision and energy transfer between the protein excited state molecule and the quencher molecule [29]. To further elucidate the inhibition mechanism of phenolic acids on digestive enzymes, we calculated the number of binding sites (N), the quenching constant (K*_q_*), the binding constant (K_a_), and the Stern-Volmer constant (K*_sv_*) for two types of phenolic acids (CA and p-HA) with two digestive enzymes at three different temperatures. As shown in Table 2, the value of N is close to 1, indicating that there is only one binding site between the phenolic acids and the two starch-digesting enzymes. The decrease in K_a_ with increasing temperature suggests that higher temperatures reduce the stability of the phenolic acid-digestive enzyme complexes, and the quenching of the intrinsic fluorescence of both digestive enzymes by phenolic acids is primarily static quenching [30]. At 310 K, the binding constant K_a_ for the p-HA α-amylase system (1.42 ± 0.07 10^6^L/mol) was higher than that for the CA-α-amylase system (1.03 ± 0.35 10^6^L/mol), while the K_a_ for the CA-α-glucosidase system (2.12 ± 0.28 10^6^L/mol) was greater than that for the p-HA-α-glucosidase system (1.21 ± 0.15 10^6^L/mol). According to Dai et al. [31], K_a_ represents the quenching affinity between the inhibitor and the enzyme. A higher K_a_ value indicates stronger quenching affinity and greater inhibitory ability of the inhibitor toward the enzyme. Furthermore, in dynamic quenching, an increase in temperature promotes effective molecular collisions, leading to a rise in K*_sv_*. Conversely, a decrease in K*_sv_* with increasing temperature indicates static quenching. As shown in Table 2, similar to the trend observed for K*_a_*, K*_sv_* gradually decreased with increasing temperature, further confirming the static quenching mechanism. At 310 K, the K*_sv_* value of CA was higher than that of p-HA, indicating that CA has the strongest quenching effect on the intrinsic fluorescence of α-glucosidase. In summary, compared to α-amylase, both p-HA and CA exhibit stronger affinity toward α-glucosidase, with CA demonstrating greater inhibitory ability. These findings are consistent with the IC_50_ values obtained from the inhibition efficacy experiments.

Generally, the intermolecular forces driving enzyme–ligand binding include hydrogen bonding, hydrophobic interactions, electrostatic forces, and van der Waals forces [21]. The primary driving forces for the interaction between phenolic acids and the two enzymes (α-amylase/α-glucosidase) can be determined by ΔH and ΔS. As shown in Table 2, the negative ΔG values for all systems indicate that the interactions between the two phenolic acid compounds and α-amylase/α-glucosidase occur spontaneously [31]. According to the criteria established by Ross and Subramanian, ΔH < 0 and ΔS < 0 suggest that the main forces governing the interaction between α-glucosidase and CA are hydrogen bonding and van der Waals forces. This binding process is primarily an enthalpy-driven exothermic reaction, and lowering the temperature favors their association [24]. Yang et al. [32] also reported similar findings, namely that the binding reaction between diacylated anthocyanins from purple sweet potato and α-amylase is exothermic and enthalpy-driven.

### 3.5. UV-Visible Absorption Spectra

Alterations in aromatic amino acid residues can lead to changes in the ultraviolet (UV) absorption of proteins. Therefore, UV spectroscopy serves as a useful tool for analyzing structural changes in proteins. Figure 5E–H displays the UV-Vis absorption spectra of α-amylase/α-glucosidase in the presence of phenolic acids. It can be observed that the addition of CA and p-HA to the α-amylase/α-glucosidase solution resulted in an increase in UV absorption accompanied by a red shift as the phenolic acid concentration increased. This indicates an interaction between the phenolic acids and the digestive enzymes. Such interaction-induced alterations in the microenvironment surrounding the polypeptide backbone and the aromatic amino acid residues (Trp, Tyr, and Phe) of α-amylase/α-glucosidase, signifying a conformational change in the enzyme structure [33]. Their study demonstrated that oolong tea polyphenols, EGCG, and EGCG3”Me could cause extension of the peptide bonds in proteins (enzymes) and disrupt hydrophobic interactions. Furthermore, studies indicate that static quenching can either increase or decrease a protein’s absorption spectrum, whereas dynamic quenching, affecting only the excited state of the fluorophore, does not alter the absorption spectrum [34]. Consequently, these results further confirm that the binding of CA and p-HA to α-amylase/α-glucosidase follows a static quenching mechanism, a conclusion consistent with the findings from fluorescence spectroscopy studies.

### 3.6. Circular Dichroism

**Figure 6:**
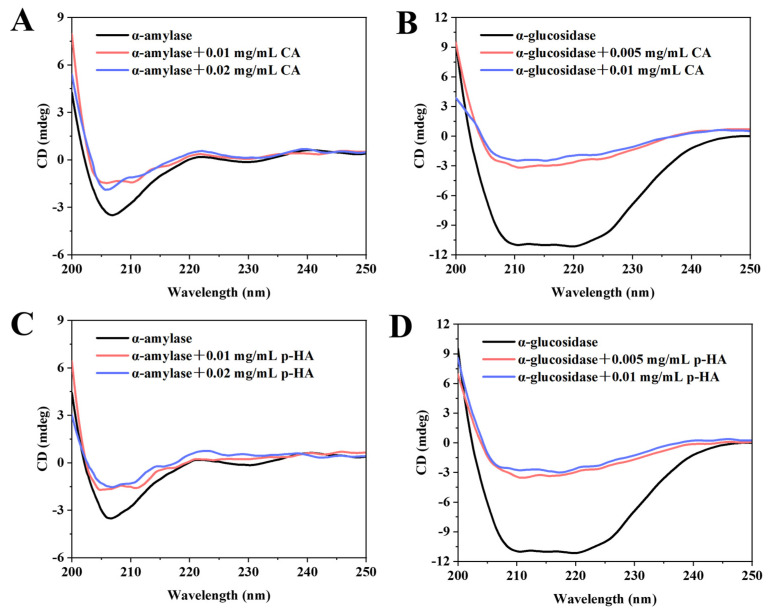
The respective effects of caffeic acid (CA) and p-hydroxycinnamic acid (p-HA) on the circular dichroism spectra of α-amylase (**A**,**C**) and α-glucosidase (**B**,**D**).

Figure 6 shows the circular dichroism (CD) spectra of α-amylase and α-glucosidase. Two characteristic negative peaks for α-helices were observed at 207 nm (π→π* transition) and 219 nm (n→π* transition) [35]. Upon addition of CA and p-HA, the CD intensities of both bands decreased systematically. This indicates that interactions between the phenolic acids and the enzymes (α-amylase/α-glucosidase) induced alterations in their secondary structure, specifically a reduction in α-helical content [30,36]. The effects on the secondary structure composition of α-amylase and α-glucosidase are detailed in Table S2. For α-amylase, the initial percentages were 16.9% α-helix, 37.9% β-sheet, 14.1% β-turn, and 34.9% random coil. Following the addition of phenolic acids, the α-helical content of α-amylase decreased (from 16.9% to 15.0% with CA and 13.7% with p-HA), while the contents of β-sheet (increasing from 37.9% to 39.2% and 40.0%), β-turn (from 14.1% to 14.6% and 16.5%), and random coil (from 34.9% to 36.1% and 36.5%) increased. Consequently, the ratio of β/α increased (from 3.077 to 3.587 and 4.124). Similarly, the addition of p-HA reduced the α-helical proportion and increased the proportions of β-sheet, β-turn, and random coil in α-glucosidase (Table S2). Integrating these findings with the results from kinetic and fluorescence spectroscopy analyses suggests that both phenolic acids likely bind to the enzymes, forming inhibitor-enzyme complexes. This binding alters the enzyme conformation (inducing folding or unfolding of specific polypeptide segments), potentially hindering the formation of a functional active site. Consequently, this impedes substrate recognition and binding, ultimately leading to enzyme inhibition [37].

### 3.7. Effects of CA and p-HA on Glucose Absorption Characteristics

Following the inhibition of starch-digesting enzymes (α-amylase and α-glucosidase) by phenolic acids, the resulting oligosaccharides and disaccharides are further hydrolyzed into glucose, which is primarily absorbed in the small intestine [38]. In order to comprehensively evaluate the regulatory effects of caffeic acid (CA) and p-hydroxycinnamic acid (p-HA) on intestinal glucose absorption *in vitro* models, we extended our investigation to evaluate their influence on intestinal glucose absorption using an *ex* *vivo* everted gut sac model. This approach allows for the direct examination of whether these compounds interfere with glucose uptake across different intestinal segments, complementing their enzymatic inhibition profile and providing a more integrated understanding of their hypoglycemic mechanisms.

**Figure 7:**
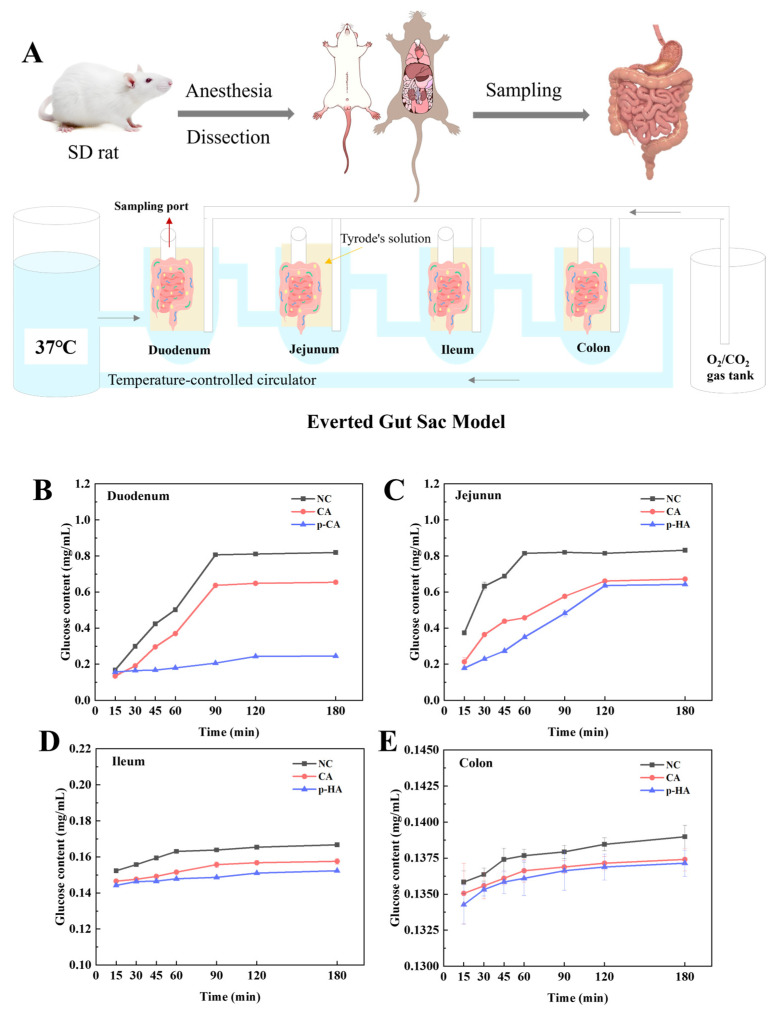
Effects of caffeic acid and p-hydroxycinnamic acid on glucose absorption in different intestinal segments-duodenum (**B**), jejunum (**C**), ileum (**D**), and colon (**E**). NC: Native control (2 mg/mL glucose); CA: Caffeic acid (2 mg/mL glucose + 1 mg/mL CA); p-HA: p-hydroxycinnamic acid (2 mg/mL glucose + 1 mg/mL p-HA).

As shown in Figure 7, basal glucose absorption (NC group) varied significantly across intestinal regions, with the highest rates observed in the duodenum (0.82 ± 0.00 mg/mL) and jejunum (0.83 ± 0.01 mg/mL), markedly exceeding those in the ileum (0.17 ± 0.00 mg/mL, *p* < 0.05) and colon (0.14 ± 0.00 mg/mL, *p* < 0.05). Treatment with CA (1 mg/mL) may significantly delay glucose absorption, most notably in the duodenum (reduction of 20.73%) and jejunum (19.28%), while no statistically significant effect was detected in the ileum or colon. Similarly, p-HA (1 mg/mL) exhibited a strong inhibitory effect on glucose absorption in both the duodenum (70.73%, from 0.82 ± 0.00 mg/mL to 0.24 ± 0.00 mg/mL, *p* < 0.05) and jejunum (22.89%, *p* < 0.05), with no notable impact in distal segments. Importantly, p-HA demonstrated a significantly stronger inhibitory effect than CA specifically in the duodenum (reduction: 70.73% vs. 20.73%, *p* < 0.05). These results indicate that both CA and p-HA effectively inhibit intestinal glucose absorption, with segment-specific efficacy concentrated in the proximal small intestine (the primary site of dietary glucose uptake). The combined evidence of enzymatic inhibition and reduced ex vivo glucose absorption supports a local intestinal mechanism for these phenolic acids in modulating carbohydrate uptake. These observations are consistent with studies reporting that inhibiting digestion and absorption in the small intestine may contribute to glycemic control [38]. The everted intestinal sac is a classic *ex vivo* model that maintains intestinal epithelial structure and basic transport function. However, it lacks in vivo blood circulation, neural regulation, and gut microbiota, and tissue viability is limited ex vivo. Thus, the present results reflect the potential effects of CA and p-HA on intestinal glucose absorption. Further in vivo studies and investigations on glucose transporters (SGLT1, GLUT2) are needed. Our findings not only reinforce the inhibitory effects of CA and p-HA on key starch-digesting enzymes but also elucidate their additional function in limiting intestinal glucose uptake. This dual mode of action enhances their potential as functional food components or nutraceuticals for glycemic regulation. Further studies are warranted to identify the molecular targets involved in transport inhibition and to validate these effects in *in vivo* models.

## 4. Discussion

In summary, this study systematically elucidates the inhibitory effects and underlying mechanisms of caffeic acid (CA) and p-hydroxycinnamic acid (p-HA) on key starch-digesting enzymes. The results demonstrate that both phenolic acids function as potent mixed-type inhibitors of α-amylase and α-glucosidase, with a marked selectivity towards α-glucosidase. A combination of multi-spectroscopic analyses and molecular docking revealed that the inhibition is mediated through a complex interaction network involving hydrogen bonding, hydrophobic forces, and π-π stacking, which subsequently induces conformational changes in the enzyme structures. Notably, the ortho-dihydroxyl configuration of CA and the para-hydroxyl group of p-HA were identified as critical structural determinants governing their binding affinity and inhibitory potency, establishing a clear structure–activity relationship. Beyond enzyme inhibition, this research provides novel mechanistic insight by demonstrating that both compounds significantly suppress glucose absorption in the proximal small intestine using an *ex vivo* model, with p-HA exhibiting exceptional efficacy in the duodenum. A novel functional contribution of this study is the demonstration that CA and p-HA exert a dual hypoglycemic effect: not only inhibiting starch digestive enzymes but also directly suppressing glucose absorption in the proximal small intestine. Notably, p-HA shows 70.73% inhibition of glucose absorption in the duodenum, which is significantly stronger than CA, revealing segment-specific and compound-specific functional differences. This integrated mechanism (enzyme inhibition + intestinal absorption inhibition) provides a more complete functional explanation for the hypoglycemic potential of hydroxycinnamic acids, going beyond the single enzyme inhibition mechanism reported in most previous studies. While these findings are derived from mechanistic *in vitro* and *ex vivo* investigations, they robustly position CA and p-HA as promising natural candidates for managing carbohydrate metabolism. This study not only deepens the understanding of phenolic acid bioactivity but also paves the way for future research, including *in vivo* validation and the development of these compounds or their derivatives as functional food ingredients for the prevention and management of metabolic disorders. Despite the potent *in vitro* inhibitory effects, the bioavailability of hydroxycinnamic acids is restricted by low water solubility and metabolism by gut microbiota. However, these compounds still act locally in the intestinal lumen to delay carbohydrate digestion and attenuate postprandial blood glucose spikes. Thus, they show potential as functional food ingredients or adjuvants for glycemic regulation.

This study is limited by its *in vitro*/*ex vivo* focus, which may not translate fully to *in vivo* settings. Bioavailability, metabolism, and safety data for the tested phenolics are absent, and only a limited range of derivatives was assessed. Future research should focus on *in vivo* models and bioavailability studies to validate the real-world potential of these compounds.

## Figures and Tables

**Table 1:** IC_50_ values of polyphenols and inhibition kinetic parameters.

	Polyphenols	Concentration (mg/mL)	IC_50_ (mg/mL)	K*_m_* (mg/mL)	V*_max_* (mg/min)	K*_ic_* (mg/mL)	K*_iu_* (mg/mL)
α-amylase	CA	0	1.32 ± 0.15	0.20 ± 0.01 ^a^	0.31 ± 0.01 ^a^	0.80 ± 0.08	2.31 ± 0.23
		1		0.17 ± 0.01 ^b^	0.24 ± 0.00 ^b^		
		1.5		0.16 ± 0.00 ^bc^	0.21 ± 0.00 ^c^		
		2		0.15 ± 0.01 ^c^	0.17 ± 0.00 ^d^		
	p-HA	0	1.12 ± 0.25	0.20 ± 0.01 ^a^	0.31 ± 0.01 ^b^	0.31 ± 0.07	1.34 ± 0.10
		1		0.10 ± 0.02 ^b^	0.36 ± 0.04 ^a^		
		1.5		0.09 ± 0.01 ^b^	0.35 ± 0.02 ^a^		
		2		0.10 ± 0.00 ^b^	0.25 ± 0.00 ^c^		
α-glucosidase	CA	0	0.07 ± 0.01	0.00 ± 0.28 ^d^	0.90 ± 1.60 ^a^	0.07 ± 0.01	0.12 ± 0.02
		1		0.99 ± 0.08 ^c^	0.16 ± 0.01 ^b^		
		1.5		4.45 ± 0.05 ^a^	0.03 ± 0.00 ^c^		
		2		4.01 ± 0.05 ^b^	0.03 ± 0.00 ^c^		
	p-HA	0	0.64 ± 0.18	0.00 ± 0.28 ^d^	0.90 ± 1.60 ^a^	0.49 ± 0.01	0.77 ± 0.03
		1		0.69 ± 0.05 ^c^	0.37 ± 0.02 ^b^		
		1.5		2.84 ± 1.07 ^b^	0.06 ± 0.02 ^c^		
		2		3.21 ± 0.82 ^a^	0.03 ± 0.01 ^c^		

These values are presented in the form of mean ± standard deviation (SD). Different letters indicate statistical differences (*p* < 0.05).

**Table 2:** Effect of CA and p-HA on the fluorescence quenching parameters and thermodynamic parameters of α-amylase and α-glucosidase.

	Polyphenols	T (K)	R^2^	K*_q_* (L/mol/S)	N	K_a_ (10^6^L/mol)	K*_sv_* (10^6^L/mol)	ΔH (kJ/mol)	ΔG (kJ/mol)	ΔS (J/mol/K)
α-amylase	CA	298	0.99	0.80 × 10^13^	0.70 ± 0.08 ^a^	1.41 ± 0.12 ^a^	0.04 ± 0.00 ^a^	−62.44 ± 0.24	−85.27 ± 8.76 ^c^	−135.10 ± 3.41
		304	0.99	0.64 × 10^13^	0.70 ± 0.03 ^a^	1.31 ± 0.01 ^ab^	0.03 ± 0.00 ^b^		−76.18 ± 7.23 ^b^	
		310	0.99	0.46 × 10^13^	0.72 ± 0.02 ^a^	1.03 ± 0.35 ^b^	0.02 ± 0.00 ^c^		−68.48 ± 3.46 ^a^	
	p-HA	298	0.99	1.28 × 10^13^	0.65 ± 0.06 ^a^	1.57 ± 0.07 ^a^	0.06 ± 0.00 ^a^	−4.74 ± 0.09	−93.75 ± 8.17 ^b^	−15.71 ± 0.83
		304	0.99	1.24 × 10^13^	0.62 ± 0.06 ^ab^	1.44 ± 1.08 ^a^	0.06 ± 0.00 ^a^		−75.79 ± 6.79 ^a^	
		310	0.99	0.80 × 10^13^	0.58 ± 0.10 ^b^	1.42 ± 0.07 ^a^	0.04 ± 0.00 ^b^		−72.88 ± 5.85 ^a^	
α-glucosidase	CA	298	0.99	2.30 × 10^13^	0.22 ± 0.05 ^a^	2.64 ± 0.32 ^a^	0.11 ± 0.03 ^a^	−119.64 ± 0.25	−20.58 ± 0.03 ^c^	−158.47 ± 5.24
		304	0.99	2.02 × 10^13^	0.31 ± 0.05 ^ab^	2.26 ± 0.21 ^ab^	0.10 ± 0.03 ^ab^		−19.48 ± 0.01 ^b^	
		310	0.99	1.80 × 10^13^	0.41 ± 0.08 ^c^	2.12 ± 0.28 ^b^	0.09 ± 0.03 ^b^		−10.19 ± 0.04 ^a^	
	p-HA	298	0.99	1.14 × 10^13^	1.19 ± 0.13 ^a^	1.63 ± 0.06 ^a^	0.06 ± 0.00 ^a^	−39.33 ± 1.00	−91.29 ± 7.57 ^c^	−159.13 ± 1.91
		304	0.99	1.08 × 10^13^	1.30 ± 0.09 ^ab^	1.39 ± 0.14 ^b^	0.05 ± 0.00 ^b^		−32.30 ± 3.48 ^b^	
		310	0.99	1.04 × 10^13^	1.53 ± 0.15 ^c^	1.21 ± 0.15 ^b^	0.05 ± 0.00 ^b^		−10.49 ± 0.43 ^a^	

These values are presented in the form of mean ± standard deviation (SD). Different letters indicate statistical differences (*p* < 0.05). K*_q_*, fluorescence quenching rate constant; N, number of binding sites; K_a_, binding constant; K*_sv_*, quenching constants; ΔH, enthalpy change; ΔS, entropy change; ΔG, gibbs free energy; CA, caffeic acid; p-HA, p-hydroxycinnamic acid.

## Data Availability

The original contributions presented in this study are included in the article/Supplementary Materials. Further inquiries can be directed to the corresponding authors.

## Supplementary Materials

The following supporting information can be downloaded at: https://www.mdpi.com/article/10.3390/foods15111972/s1. Table S1: Molecular docking of caffeic acid (CA) and p-hydroxycinnamic acid (p-HA) on α-amylase and a-glucosidase. Table S2: Effect of CA and p-HA on the secondary structure content of α-amylase and α-glucosidase.
