# Supplementary material for: Molecular Basis for the Divergent Inhibition of α-Amylase and α-Glucosidase by Phenolic Acids: The Critical Role of Hydroxyl Substitution
Source: Foods. 2026 Jun 2;15(11):1972. doi: 10.3390/foods15111972 (PMC13256135; doi:10.3390/foods15111972)
Supplement: Supplementary file 1 [file foods-15-01972-s001.zip › foods-4259511-supplementary.pdf]

Supplementary material

Molecular basis for the divergent inhibition of α-amylase and α-glucosidase by phenolic acids: the critical role of hydroxyl substitutiontle

Shuang Yang [1,2], Yongxing Li [2], Weiyu Han [2], Wenhao Cao [2], Zhihui Hu [2], Zhangliang Zhu [3], Mei Li [2,*], Jianhui Feng[2,*], Jinfang Zhang [1,*]

1 School of Food Engineering, Ludong University, Yantai 264025, China; syang979797@163.com (S.Y); jfzhang1007@163.com (J. Z)

2 Center for Mitochondria and Healthy Aging, College of Life Sciences, Yantai University, Yantai 264005, China; syang979797@163.com (S.Y); liyongxing0826@163.com (Y. L); hwy02282024@163.com (W.H); wenhaocao2024@163.com (W.C); huzhihui9968@163.com (Z.H); jhfeng0122@163.com (J. F); lmei9206@163.com (M.L)

3 Graduate School of Biostudies, Kyoto University, Kyoto 606-8501, Japan; zhu.zhangliang.8i@kyoto-u.ac.jp (Z. Z)

* Correspondence: lmei9206@163.com (M.L)

Table S1. Molecular docking of caffeic acid (CA) and p-hydroxybenzoic acid (p-HA) on α-amylase and a-glucosidase.

Compounds	The residues of hydrogen bonding	Number of hydrogen bonds	Binding Energy
α-amylase - CA	Gln63 Glu233 Asp197	5	-6.5
α-amylase - p-HA	Gln63 Asp197	2	-6.2
α-glucosidase - CA	Asp382 Asp289 Gly286 Lys290	4	-5.4
α-glucosidase - p-HA	Glu180	1	-5.3

Table S2. Effect of CA and p-HA on the secondary structure content of α-amylase and α-glucosidase.

	α-amylase						α-glucosidase					
Polyphenols	Concentration (mg/mL)	α-Helix (%)	β-Sheet (%)	β-Turn (%)	Random coil (%)	β/α	Concentration (mg/mL)	α-Helix (%)	β-Sheet (%)	β-Turn (%)	Random coil (%)	β/α
CA	0	16.9	37.9	14.1	34.9	3.077	0	95.3	0.2	3.9	0.3	0.043
	0.01	16.5	38.4	14.4	35.7	3.200	0.005	89.1	1.9	3.3	6.2	0.058
	0.02	15.0	39.2	14.6	36.1	3.587	0.01	88.6	2.5	4.1	7.4	0.074
p-HA	0	16.9	37.9	14.1	34.9	3.077	0	95.3	0.2	3.9	0.3	0.043
	0.01	14.4	39.9	15.2	35.3	3.826	0.005	94.0	0.4	4.7	0.9	0.054
	0.02	13.7	40.0	16.5	36.5	4.124	0.01	93.3	0.8	8.8	1.8	0.103
